# Emergency Total Thyroidectomy for Bleeding Anaplastic Thyroid Carcinoma: A Viable Option for Palliation

**DOI:** 10.4103/0973-1075.78452

**Published:** 2011

**Authors:** Sunil Kumar, Mohit Kumar Joshi

**Affiliations:** Department of Surgery, University College of Medical Sciences and Guru Teg Bahadur Hospital, Delhi, India

**Keywords:** Anaplastic thyroid carcinoma, Thyroidectomy, Active bleeding

## Abstract

Anaplastic thyroid carcinoma (ATC) is a rare and highly aggressive thyroid neoplasm. Bleeding from tumor is an uncommon, but potentially life-threatening complication requiring sophisticated intervention facilities which are not usually available at odd hours in emergency. We report the case of a 45-year-old woman who presented with exsanguinating hemorrhage from ATC and was treated by emergency total thyroidectomy. The patient is well three months postoperatively. Emergency total thyroidectomy is a viable option for palliation in ATC presenting with bleeding.

## INTRODUCTION

Anaplastic thyroid carcinoma (ATC) is a rare but rapidly fatal disease, owing to extensive locoregional spread and early distant metastasis. Most patients die of upper airway obstruction, with hemorrhage as another known cause of fatality. Management of emergent and life-threatening external bleeding from ATC is challenging and reported scarcely in the literature. It usually requires resources and expertise which may not be available in an average setup in a developing country, especially in emergency situations. We report our solitary experience of performing emergency total thyroidectomy for external bleeding from ATC and propose it as a modality for palliation of this fatal complication.

## CASE REPORT

**Figure 1:**
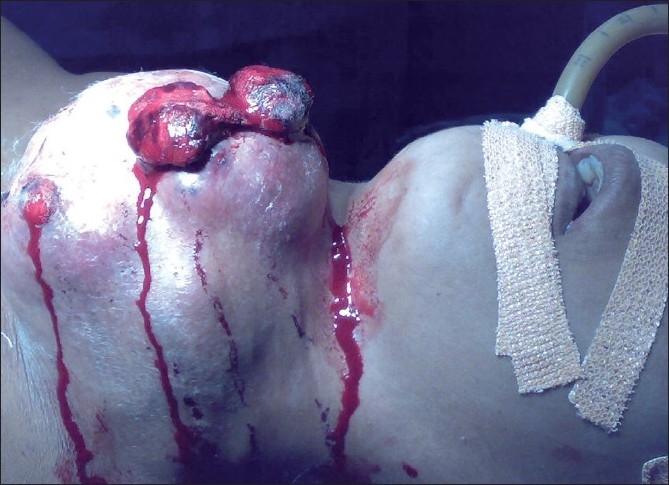
Thyroid mass with active bleeding

**Figure 2:**
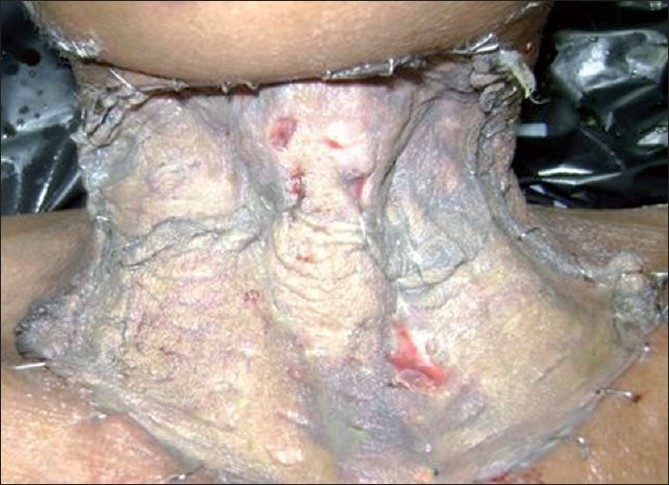
Raw area following thyroidectomy covered with skin graft

A 45-year-old woman presented to surgical emergency with profuse bleeding from a mass in the front of the neck. The mass, present since one and half years, had rapidly increased in size over two months and had started bleeding 2 h prior to hospitalization. On examination, she was conscious, oriented, pale, and dehydrated with a low volume pulse of 120 beats/ min and blood pressure of 90/70 mmHg. Local examination revealed an ulcero-proliferative growth of 12 x 8 cm in anterior part of neck with multiple bleeding points [Figure 1]. She was resuscitated with IV fluids and three units whole blood transfusion. Written consent for anesthesia and surgery including that for death on table was obtained and broadspectrum antibiotics were started. The patient was placed in thyroidectomy position and a long elliptical incision was made. A large blood clot was found in subcutaneous plane. Following clot evacuation, three pulsating bleeding points were seen which were controlled by suture ligation. Deeper dissection revealed the mass to be adhered to the left common carotid artery, trachea and esophagus. The mass was dissected free from these structures, although small bits of tumor which were inseparably adherent could not be removed. Bared carotid arteries and internal jugular veins were covered by flaps of residual sternocleidomastoid muscle. The large skin defect was covered by skin graft taken from the right thigh [Figure 2]. The histologic diagnosis was ATC. Postoperative course was uneventful. A chest X-ray and ultrasonography of abdomen, done 10 days after surgery, revealed no pulmonary or hepatic metastasis. Treatment with combination of external beam radiotherapy (EBRT) (60.8 Gy in 32 fractions over 21 days) and chemotherapy (doxorubicin and cisplatin) was begun 4 weeks postoperatively. The patient is disease-free 3 months postoperatively.

## DISCUSSION

ATC is rare and comprises only 1%-3% of all thyroid neoplasm. It is one of the most aggressive, lethal and difficult malignancies to treat, with very high mortality rates. Median survival is 4-5 months from the time of diagnosis. Majority die of aggressive loco-regional disease, causing upper airway obstruction.[1] Other causes of fatality include circulatory failure, hemorrhage, and respiratory insufficiency.

Hemorrhage accounts for 15% mortality.[2] Since external bleeding is a rare complication, a universally agreed treatment modality to deal with it does not exist. Selective embolization of thyroid arteries (SETA) is a safe and minimally invasive method for palliation of ATC; it can also be used to control intractable hemorrhage.[3] Successful treatment of thyroid artery erosion by esophageal carcinoma using superselective catheterization and embolization of the bleeding vessel has also been reported.[4] EBRT has also been used with success for achieving hemostasis in various malignancies including carcinoma of head and neck, lung, skin, vagina, rectum, and urinary bladder.[5] Radiation causes sclerosis of vessels, thus promoting hemostasis. Although the dose of hemostatic radiotherapy differs depending on the site affected, the optimal dose and fractionation remains controversial. It is widely agreed that lower total doses with larger daily fractions over short time achieves hemostasis better with less side effects.[6] As SETA and EBRT were not available to us in the emergency, we could not use these in our patient. Recurrent laryngeal nerve may either be involved by tumor or damaged during difficult thyroidectomy, written consent for tracheostomy must be obtained and the surgeon should be prepared for the same.

This is the first human case report where total thyroidectomy has been performed to treat actively bleeding ATC, although one such case has been reported in a dog by Slensky *et al*.[7] Earlier, thyroidectomy has been performed to deal with post-traumatic bleeding from thyroid gland.[8,9] As radiation is better tolerated by myocutaneous flaps compared to skin grafts, if possible the raw area should be covered by myocutaneous flaps.

## CONCLUSIONS

Total thyroidectomy is a viable option for palliation in patients presenting with actively bleeding ATC if hemostatic radiotherapy and SETA are unavailable.
